# Distinct mechanisms survey the structural integrity of HLA-B*27:05 intracellularly and at the surface

**DOI:** 10.1371/journal.pone.0200811

**Published:** 2018-08-02

**Authors:** Zeynep Hein, Britta Borchert, Esam Tolba Abualrous, Sebastian Springer

**Affiliations:** 1 Department of Life Sciences and Chemistry, Jacobs University, Bremen, Germany; 2 Department of Mathematics and Computer Science, Freie Universität, Berlin, Germany; Saint George's University, UNITED KINGDOM

## Abstract

HLA-B*27:05 is associated with the development of autoimmune spondyloarthropathies, but the precise causal relationship between the MHC haplotype and disease pathogenesis is yet to be elucidated. Studies focusing on the structure and cellular trafficking of HLA-B*27:05 implicate several links between the onset of inflammation and the unusual conformations of the molecule inside and at the surface of antigen presenting cells. Several lines of evidence emphasize the emergence of those unnatural protein conformations under conditions where peptide loading onto B*27:05 is impaired. To understand how cellular factors distinguish between poorly loaded molecules from the optimally loaded ones, we have investigated the intracellular transport, folding, and cell surface expression of this particular B27 subtype. Our findings show that B*27:05 is structurally unstable in the absence of peptide, and that an artificially introduced disulfide bond between residues 84 and 139 conferred enhanced conformational stability to the suboptimally loaded molecules. Empty or suboptimally loaded B*27:05 can escape intracellular retention and arrive at the cell surface leading to the appearance of increased number of *β*_2_m-free heavy chains. Our study reveals a general mechanism found in the early secretory pathways of murine and human cells that apply to the quality control of MHC class I molecules, and it highlights the allotype-specific structural features of HLA-B*27:05 that can be associated with aberrant antigen presentation and that might contribute to the etiology of disease.

## Introduction

Major histocompatibility complex (MHC) class I molecules are transmembrane glycoproteins playing a key role in the anti-viral and anti-tumor immune response, by presenting antigenic peptides to cytotoxic T lymphocytes, which then kill aberrant cells. The assembly of the class I heavy and light chains (beta-2 microglobulin, *β*_2_m) as well as the antigenic peptide ligand takes place in the endoplasmic reticulum (ER). The binding of the peptide involves the concerted action of various proteins forming the peptide loading complex (PLC): the lectin chaperone calreticulin, binding to the N-linked glycans of the heavy chain; the disulfide isomerase ERp57, triggering disulfide bonding; the transporter associated with antigen processing (TAP), channeling the peptides generated in the cytosol into the ER lumen; and the class I-specific chaperones tapasin and TAPBPR, which are involved in peptide editing [1–3]. Binding of a high-affinity peptide to the class I-*β*_2_m heterodimer is decisive for the further fate of the complex inside the cell, since suboptimally loaded class I molecules (bound to low-affinity peptides or possibly even devoid of any peptide) do not reach the cell surface under normal conditions but are retained in a cycle between the compartments of the early secretory pathway (ER, ER-Golgi intermediate compartment (ERGIC), and the cis-Golgi) instead [4–6]. Another quality control takes place at the plasma membrane, where class I molecules eventually lose their peptide and *β*_2_m, causing endocytic destruction of the heavy chain via its transport to degradative compartments [7,8].

Comparative molecular dynamics (MD) simulations on empty and peptide-bound class I molecules have shown an increased flexibility of the empty peptide binding domain, especially around the F pocket where the peptide C terminus binds [9,10]. Fluctuations in this region might be recognized by cellular quality control mechanisms. Indeed, the murine class I molecule H-2D^b^ was stabilized with only a partial ligand corresponding to the C-terminal portion of an optimal peptide [11]. Further molecular dynamics and experimental studies have confirmed the influence of the peptide C terminus on stabilizing the F pocket region, which dominates the conformational and thermodynamic properties of the protein and is surveyed by the cellular quality control [12]. In agreement with these findings, folded conformation of the F pocket of the murine class I molecule H-2K^b^ can be preserved in a peptide-independent manner by introducing an artificial disulfide bond resulting in restricted mobility of this region [13]. As a result, the disulfide mutant can reach the cell surface in its suboptimally loaded state and shows an increased half-life there, thus circumventing the quality control mechanisms mentioned above.

The human leukocyte antigen HLA-B27 is one of the most prominent and thoroughly studied MHC class I molecules due to the genetic association of some of its subtypes, such as B*27:05 and B*27:04 with ankylosing spondylitis (AS) and acute anterior uveitis [14–16]. While the molecular mechanisms underlying AS pathogenesis remain unclear, studies emphasize various lines of possibilities. Interestingly, while a major line of evidence point to altered peptide presentation via AS-related B27 subtypes [17], induction of inflammation by non-conventional B27 structures (i.e. disulfide-linked B27 heavy chains) in case of poor peptide loading has also been suggested [18].

We have previously attempted to understand the molecular features of B27 subtypes that confer susceptibility to AS by comparing disease-related subtype B*27:05 with the disease-unrelated B*27:09. MD simulations and *in vitro* analyses revealed increased molecular disorder in the alpha helices involved in the F pocket region in the 05 subtype compared to the 09, leading to a general instability of the heavy chains of B*27:05 [12]. In order to test whether B*27:05 can be conformationally stabilized by limiting the mobility of this region, we constructed an F pocket disulfide mutant of B*27:05 by linking residues 84 and 139 with a disulfide bond, and we investigated its cellular maturation and behavior at the cell surface. Our analyses reveal that the A139C/Y84C mutant (hereafter referred to as B*27:05-Y84C) has increased thermal stability and *β*_2_m affinity, and that it can reach the cell surface in the absence of optimal peptides. This confirms the importance of the F pocket for MHC class I quality control in cells and demonstrate that this region can artificially be stabilized not only in murine but also in human class I allotypes.

## Materials and methods

### Cell lines, antibodies and peptides

Human TAP2-deficient STF1 cells [19,20] were maintained using standard protocols. The TAP2-reconstituted cell line STF1-TAP2 was generated by introducing the human *TAP2* gene into the STF1 cells by viral transduction as described previously [13]. We used the monoclonal antibodies W6/32, a conformation-specific anti-HLA antibody [21], BBM.1, human *β*_2_m-specific antibody [22], and anti-HA (influenza virus hemagglutinin) antibody (clone 12CA5). For the detection of TAP2, a monoclonal antibody (429.3) was used (kind gift from Peter van Endert, INSERM, Paris, France). Calnexin was detected using the polyclonal rabbit antiserum produced and kindly provided by David B. Williams (University of Toronto, Toronto, Canada). AlexaFluor 488 (AF488)-conjugated goat anti-mouse immunoglobulin G was purchased from Dianova (Hamburg, Germany). Immunofluorescence microscopy was performed as described previously [12]. Sequences of all peptides are given in the standard single-letter amino acid code. The high-affinity peptide (origin: human Cathepsin A) IRAAPPPLF and IRAAPPPK(Biotin)F were synthesized by Genecust (Ellange, Luxembourg), purified by HPLC to >90% (W/W).

### Plasmids and retroviral expression

The A139C and Y84C mutations and the HA epitope tag (YPYDVPDYA) were sequentially introduced into the wild-type HLA-B*27:05 sequences by QuikChange site-directed mutagenesis (Agilent). Constructs were subcloned into pEGFP-N1 via *Xho*I and *Bam*HI and used in fluorescence microscopy experiments. The N-terminally HA-tagged HLA-B*27:05 expression construct (wild type and Y84C) were also cloned into puc2CL6IPwo *via* the *Xho*I and *Bam*HI sites. Lentiviruses were produced and used for gene delivery as described previously [23]. In all experiments (except for microscopy, where N-terminal HA and C-terminal GFP double tagged versions were used), B27 constructs that were only tagged with HA at their N termini were used.

### Flow cytometry

Cells were harvested via trypsinization, fixed in sodium azide (0.02% in PBS), and stained with 12CA5 against the HA epitope tag or with W6/32 against HLA and were kept on ice. After washing, antibody-antigen complexes were labeled with secondary antibody conjugated with AlexaFluor488. Surface staining was recorded by a CyFlow®Space flow cytometer (Sysmex-Partec, Norderstedt, Germany) and analyzed by Flowjo, LLC software. Presented histograms depict fluorescence measurements from intact cells that were gated according to their forward and side light scattering properties.

### Metabolic labeling and pulse-chase analysis

In pulse-chase and thermostability analyses, newly synthesized HLA-B*27:05 molecules were labeled with ^35^S-labeled methionine and -cysteine (EasyTag EXPRESS-^35^S protein labeling mix, PerkinElmer) as described previously [24]. Briefly, cells were pulse labeled for 7 min and chased in complete growth medium containing excess amounts of unlabeled methionine and cysteine for 30, 60, 120, and 240 min, respectively. At the end of the chase period, cells were lysed in native lysis buffer (50 mM Tris·Cl, pH 7.5, 150 mM NaCl, 5 mM EDTA, 1% (v/v) Triton X-100) for one hour and folded HLA molecules were immunoprecipitated with W6/32 coupled to Protein A-agarose beads (Calbiochem, Merck Millipore). Beads were washed twice with wash buffer (50 mM Tris·Cl, pH 7.5, 150 mM NaCl, 5 mM EDTA, 0.1% (v/v) Triton X-100) and boiled at 95°C for 10 min in 50 μl denaturation buffer containing 1% sodium dodecyl sulfate (SDS) and 10 mM dithiothreitol (DTT). The supernatant was equilibrated in 1 mL wash buffer and HA-tagged HLA-B*27:05 molecules were re-immunoprecipitated with anti-HA coupled to Protein A-agarose beads. Immune complexes were washed once with wash buffer and treated with Endoglycosidase F1 as described [24].

### Western blot analysis

Cell lysates or immune complexes were resolved on 11% sodium dodecyl sulfate (SDS)- polyacrylamide gels and transferred onto polyvinylidene fluoride (PVDF) membranes. Proteins were detected by incubating the membranes with primary and alkaline phosphatase-conjugated secondary antibodies, respectively. Protein-antibody complexes were visualized by 5-bromo-4-chloro-3-indolyl-phosphate (BCIP)/nitro blue tetrazolium (NBT) colorimetric method. For non-reducing gel electrophoresis, cells were incubated with 20 mM solution of the alkylating agent N-ethylmaleimide (NEM) (Applichem, Darmstadt, Germany) prior to and during lysis [25]. Immunoprecipitation was performed as described above and immune complexes were denatured by boiling in the presence of SDS (1%) and dithiothreitol (DTT), where indicated. To prevent in-gel reduction of disulfide bonds, sample buffer was supplemented with NEM. In order to quantify the amount of *β*_2_m-bound B27 heavy chains in STF1 and STF1-TAP2 cells, we immunoprecipitated the heterodimeric complexes using mouse monoclonal anti-human *β*_2_m antibody BBM.1. Amount of B27 heavy chains that were readily associated and co-immunoprecipitated with *β*_2_m were analyzed in immunoblotting using anti-HA antibody. To prevent the dissociation of suboptimally loaded B27 molecules from *β*_2_m in STF cells, 20μM of IRAAPPPLF peptide was added to the cells immediately preceding the addition of lysis buffer. For specific peptide binding assay, we have added the IRAAPPPK(Biotin)F peptide to the post-nuclear supernatants of STF cells that were lysed with the native lysis buffer containing Triton X-100 (1%). Biotinylated proteins were recovered by neutravidin-agarose beads (ThermoFisher, Scientific, Waltham, MA, USA). Beads were boiled and associated proteins were resolved in SDS-PAGE. B27 heavy chains were detected using anti-HA antibody in immunoblotting.

### Thermostability assay

Cells were labeled with ^35^S-methionine and -cysteine for 7 min as described above and elsewhere. Post-nuclear supernatant (lysate) was kept either on ice or in a heat block at different temperatures (25, 30, 35, 40°C, respectively) for 10 min. Folded HLA molecules were immunoprecipitated with W6/32. Next, HA-tagged HLA-B*27:05 molecules were isolated from the supernatant of boiled W6/32 conjugated Protein A beads as described above. The total amount of W6/32-reactive HLA-B*27:05 molecules in the heat-treated lysates was determined by quantifying the ^35^S signal from the individual samples run on SDS-polyacrylamide gels.

### Molecular dynamics (MD) simulations

Coordinates of HLA-B*27:05 bound to the high affinity peptide IRAAPPPLF were taken from a 1.85 Å resolution X-ray crystal structure (PDB ID: 3BP4 [26]). The empty structure was prepared by deleting the corresponding peptide residues from the crystal structure. To model the disulfide mutant, the residues Y84 and A139 were mutated to cysteine residues and a disulfide bond was build using the LEAP program in Amber 12 [27]. PROPKA 3.1 [28] was used to determine protonation states at pH 7. Each complex was placed in an octahedral TiP3 [29] water box together with ten Na^+^ and Cl^−^ ions and neutralized with eight counter-ions. The structures were then subjected to 1500 steps of steepest-descent (SD) energy minimization, positionally restrained (25 kcalmol^−1^ Å^−2^), and heated from 100 to 300 K. The restraints were resolved in five steps, and then each complex was equilibrated for 1 ns and simulated for two independent 50 ns MD simulations. Short-range non-bonded interactions were taken into account up to a cut-off value of 9 Å. Long-range electrostatic interactions were treated with the particle mesh Ewald method [30]. The RMSF was measured with the ptraj program in Amber 12. Visualization of trajectories and preparation of figures were performed using Pymol [31].

### Brefeldin A decay

The Brefeldin A (BFA) decay was performed as described previously [8]. BFA was purchased from AppliChem (Darmstadt, Germany) and used at 10 μg/ml. Briefly, the cells were kept at 25°C overnight and were moved to 37°C for 30, 60, 120, and 240 min after addition of BFA. At the end of the incubation times, cells were harvested by trypsinization and stained according to the flow cytometry protocol. For the initial time point, cells kept at 25°C overnight were used.

## Results

### Disulfide mutant shows restricted mobility of the F-pocket

**Fig 1:**
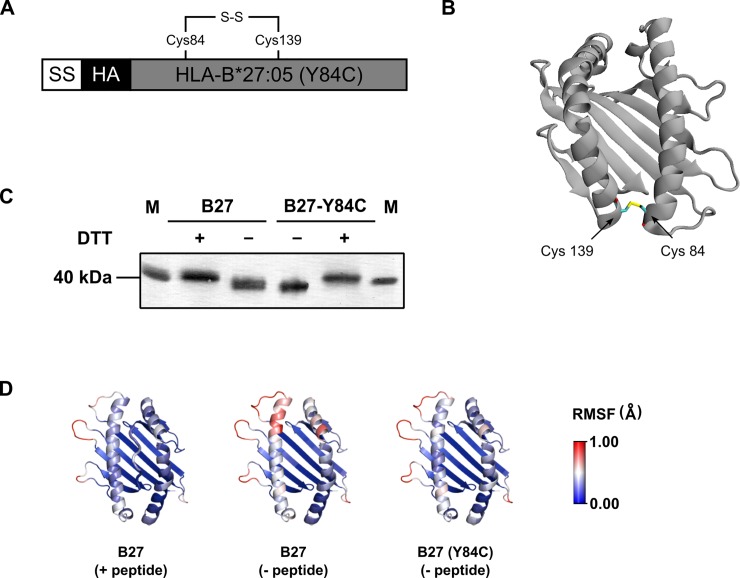
Design and and analysis of molecular flexibility of HLA B*27:05 disulfide mutant. (A) Schematic representation of the HLA B*27:05 disulfide mutant expression construct (B*27:05-Y84C). A lentiviral expression plasmid containing HLA B*27:05 was modified to introduce two cysteine codons at positions 84 and 139 (after signal sequence cleavage) to introduce a disulfide bond in the B*27:05 heavy chain. Antibody tag (HA) was introduced to the N terminus following the signal sequence (SS). (B) Modeling and schematic representation of the 84–139 disulfide bond. Ribbon diagram of B*27:05-Y84C (only residues 1 to 182 shown, peptide omitted from the image) indicates to two cysteines (represented as sticks) at positions 84 and 139 (arrows) located at α1 and α2–1 domains of the heavy chain, respectively. The structure was predicted by Disulfide by Design v2.12 [32] from the original PDB file 1HSA. (C) Confirmation of disulfide bond formation. Lysates of STF1-B*27:05 cells were subjected to immunoprecipitation with anti-HA, and the proteins were separated under reducing (+ DTT) and non-reducing (- DTT) conditions. The proteins were visualized using anti-HA antibody in immunoblotting. In the absence of DTT, B*27:05-Y84C (lane 4) runs slightly faster than wild type B*27:05 (lane 3) due to the additonal disulfide bond that renders the protein more compact. First and last lanes show the 40 kDa protein from the size marker. (D) Flexibility of the HLA B*27:05 binding groove in MD simulations. A peptide-free and 84–139 disulfide linked versions of HLA B*27:05 were derived from the crystal structure of wild type B*27:05 complexed with the cathepsin A-derived high-affinity peptide IRAAPPPLF (PDB: 3BP4). The flexibilities of the peptide binding regions were calculated as the root mean square fluctuation (RMSF) of the individual alpha carbon atoms for molecular dynamics simulations that were run for 250 ns. Values are depicted as a blue to red color spectrum on cartoon representations. In the vicinity of the C84-C139 disulfide bond, the dynamics of empty B*27:05-Y84C (right) equal those of peptide-bound wild type B*27:05 (left) while empty B*27:05 shows a higher flexibility in that region (middle).

Connecting the distal end of the peptide binding domain of murine class I allotype H-2K^b^ via C84-C139 disulfide bond improves the conformational stability of the peptide-receptive class I-*β*_2_m heterodimer [13]. Based on this finding, we wanted to know if the same disulfide modification would help another class I molecule HLA-B*27:05 to preserve its structure in the absence of high-affinity peptides. In analogy to the previously published disulfide mutant of H-2K^b^, we introduced two cysteines at positions 84 (Tyr) and 139 (Ala) of the HLA-B*27:05 coding sequence (Fig 1A). Additionally, an influenza hemagglutinin (HA) epitope tag was attached to the extracellular domain (N terminus). Formation of the disulfide bond is expected to connect the ends of alpha 1 and alpha 2–1 helices of the B*27:05 heavy chain (Fig 1B). We verified the presence of the additional (third) disulfide bond by detecting the downward shift in the migration pattern of the non-reduced B*27:05-Y84C heavy chain in gel electrophoresis (Fig 1C). We then examined the structural changes occuring in the peptide binding regions of peptide-bound and empty wild type B*27:05 and empty B*27:05-Y84C by molecular dynamics (MD) simulations. In wild type B*27:05, deletion of the peptide resulted in increased flexibility of the binding region especially in the helices flanking the F-pocket (Fig 1D, middle), whereas empty B*27:05-Y84C (right) was equally stable as wild type B*27:05 complexed with peptide (left). We conclude that the additional C84-C139 disulfide bond confers higher molecular order to the B*27:05 molecule even in the absence of high-affinity peptides.

### The disulfide mutant of HLA-B*27:05 reaches the cell surface in TAP2-deficient cells

**Fig 2:**
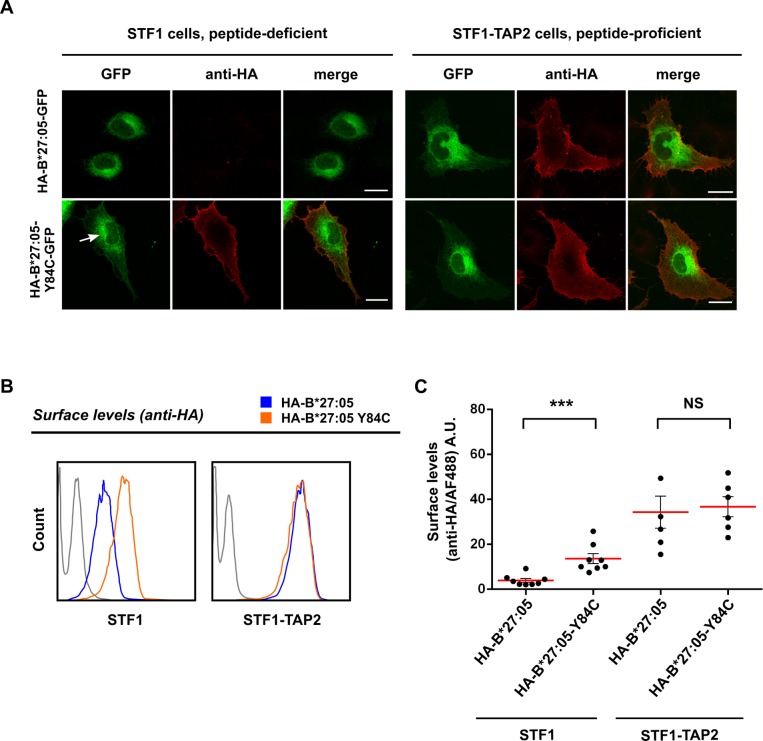
The disulfide mutant of HLA B*27:05 travels to the cell surface in TAP2-deficient cells. (A) Cell surface expression of wild type B*27:05 and B*27:05-Y84C by microscopy. STF1 and STF1-TAP2 cells were transiently transfected with C-terminally GFP-tagged B*27:05 and B*27:05-Y84C. Cells were fixed and stained using monoclonal anti-HA antibody and anti-mouse IgG conjugated with Cy3, sequentially, and observed with a confocal laser scanning microscope. In TAP2-deficient (and hence peptide-deficient) STF1 cells, wild type B*27:05 was primarily found in the tubular ER (green), and no surface stain was detected with anti-HA (red, upper row, left), whereas B*27:05-Y84C was located in the ER, the Golgi (arrow), and at the cell surface (left, lower row) and displayed strong surface stain with anti-HA antibody. In peptide-proficient STF1-TAP2 cells, the constructs were both distributed similarly, accompanied by strong cell surface HA signals (right). (B) Cell surface expression of wild type B*27:05 and B*27:05-Y84C by flow cytometry. Cells expressing either B*27:05 (blue) or B*27:05-Y84C (orange) were stained with anti-HA and anti-mouse IgG conjugated with AlexaFluor-488 and subjected to flow cytometry. Surface staining intensities of each cell line are displayed as histograms. The grey curve in both histograms shows the background signal obtained without using primary antibody. (C) Comparison of cell surface expression of wild type B*27:05 and B*27:05-Y84C in STF1 and STF1-TAP2 cells. The scatter plot (mean ± standard deviation, n = 8, n = 6) shows the distribution of individual measurements (black dots) of cell surface staining intensities of B*27:05 and B*27:05-Y84C in STF1 and STF1-TAP2 cells (***: Significant difference, P<0.001, two-tailed t-test, NS: not significant). In TAP2-deficient STF1 cells, surface expression of B*27:05-Y84C was about five times higher than for the wild type construct (left), but this difference nearly disappeared in TAP2-proficient cells (right).

We compared the cell surface expression of HA-tagged wild type B*27:05 and its disulfide mutant, B*27:05-Y84C, with regard to MHC I-specific peptide supply. From STF1 cells, which are TAP2- and hence peptide-deficient, we generated the peptide-proficient cell line STF1-TAP2 by lentiviral introduction of the human *TAP2* gene. The presence the TAP2 protein was confirmed in Western blotting (S1A Fig), and its functionality was verified by elevated endogenous HLA cell surface levels (S1B Fig). We then introduced GFP- and HA-tagged forms of wild type B*27:05and B*27:05-Y84C in STF1 and STF1-TAP2 cells and examined cell surface expression of both constructs by fluorescence microscopy and flow cytometry. Hardly any cell surface wild type B*27:05 could be detected after staining with anti-HA, while reconstitution of human *TAP2* led to an approximately ten-fold increase in wild type B*27:05 cell surface expression (Fig 2A, upper row). Cell surface expression of the B*27:05-Y84C, in contrast, was independent of TAP2 function and peptide supply, since strong cell surface signals could be observed in both TAP2-proficient and TAP2-deficient STF1 cells (Fig 2A, lower row). Flow cytometry data obtained by staining the cells with anti-HA confirmed the strong dependence of wild type B*27:05 cell surface expression on optimal peptide supply, while the cell surface expression of B*27:05-Y84C was not impaired in TAP2-deficient STF1 cells (Fig 2B and 2C). Repetition of the flow cytometry experiment with the anti-HLA monoclonal antibody W6/32 revealed the same results (S1C and S1D Fig), which parallel the increase in the surface expression of endogenous HLA Class I molecules in non-B27 transformed STF1-TAP2 cells (S1B Fig). We thus conclude that B*27:05-Y84C can overcome the anterograde intracellular quality control and reach the cell surface in the absence of peptides supplied by the TAP transporter.

### Wild type and disulfide mutant of HLA-B*27:05 are equally stable at the surface of TAP-deficient cells

**Fig 3:**
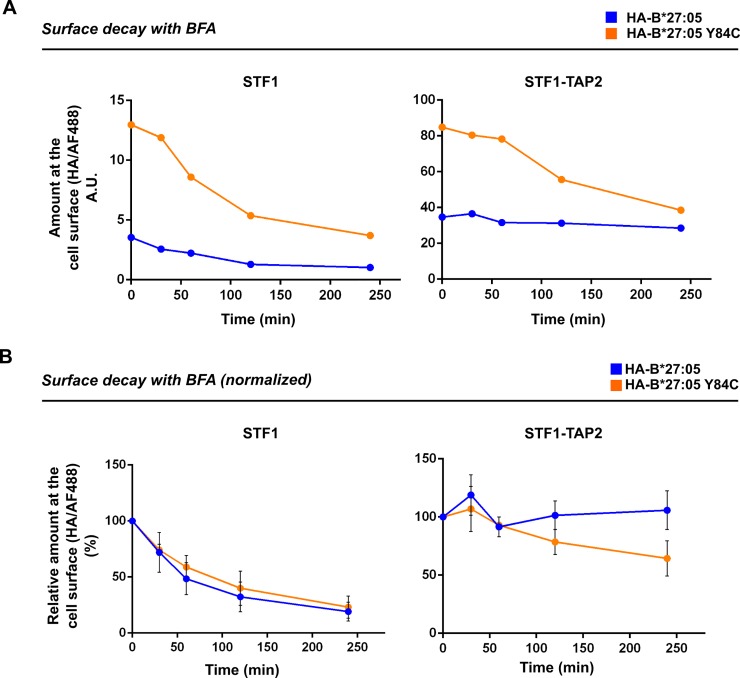
Wild type and disulfide mutant of HLA B*27:05 are equally stable at the cell surface. (A) Brefeldin (BFA) A decay. Cell surface levels of B*27:05 and B*27:05-Y84C were detected by staining STF1 (left) and STF1-TAP2 cells (right) with anti-HA. Cells were harvested and stained at the times indicated, representing the duration of treatment with BFA. For each sample, the mean fluorescence intensity value recorded by the flow cytometer is depicted on the graph. (B) Averaged decay from the cell surface. The graph shows the average cell surface levels normalized to the values detected at time point zero (SEM, n = 4). The value for time point zero was set to 100% and the following values were depicted as its percentage. In peptide-deficient STF1 cells (left), both constructs show similar cell surface lifetimes, whereas in peptide-proficient STF1-TAP2 cells (right), B*27:05-Y84C is slightly less stable at the cell surface than the wild type protein.

We have previously shown that stabilization of the F pocket via the C84-C139 disulfide linkage leads to a dramatic increase in the surface lifetime of H-2K^b^ in TAP-deficient cells [13]. In the light of those findings, we wanted to find out if the increased levels of B*27:05-Y84C at the cell surface were also due to the resistance of the stabilized molecule to endocytic degradation. For this, we followed the disappearance of both HA-tagged wild type B*27:05 and B*27:05-Y84C from the cell surface within four hours after Brefeldin A (BFA) addition to the cells. At 37°C, levels of wild type B*27:05 are very low at the surface of TAP2-deficient cells (Fig 2). To generate a sizeable pool of molecules at the cell surface, we therefore incubated the cells at lower temperature (25°C) overnight [33]. This treatment led to a nearly fourfold increase in the surface levels of wild type B*27:05 (S2A Fig). At the beginning of the experiment, B*27:05-Y84C still showed significantly higher cell surface levels than the wild type in TAP2-deficient cells (Fig 3A, left) since B*27:05-Y84C levels also increased after the 25°C incubation (data not shown). The surface signal decreased drastically over time for both variants, although B*27:05-Y84C cell surface levels after four hours of decay (time point 240 min) still slightly exceeded those of the wild type before BFA treatment (time zero). To better compare the data for wild type B*27:05 and B*27:05-Y84C, we normalized all measurement values to the values detected at time point zero and found that both constructs disappeared from the cell surface at similar rates in TAP2-deficient STF1 cells (Fig 3B, left). In peptide-proficient STF1-TAP2 cells, both variants were found to be stable at the cell surface for at least four hours (Fig 3A, right), but the B*27:05-Y84C signal started to decrease after one hour of decay and went down to approximately 70% of the starting level at time point 240 min, indicating that the disulfide mutant has a reduced lifetime at the cell surface compared to the wild type protein (Fig 3B, right). We conclude that while B*27:05-Y84C is more abundant than the wild type B*27:05 at the cell surface at any time, increased molecular stability of the B*27:05-Y84C mutant does not contribute to the dynamics of B*27:05 endocytosis.

### Disulfide-linked HLA-B*27:05 traffics faster than the wild type form in TAP2-deficient cells

**Fig 4:**
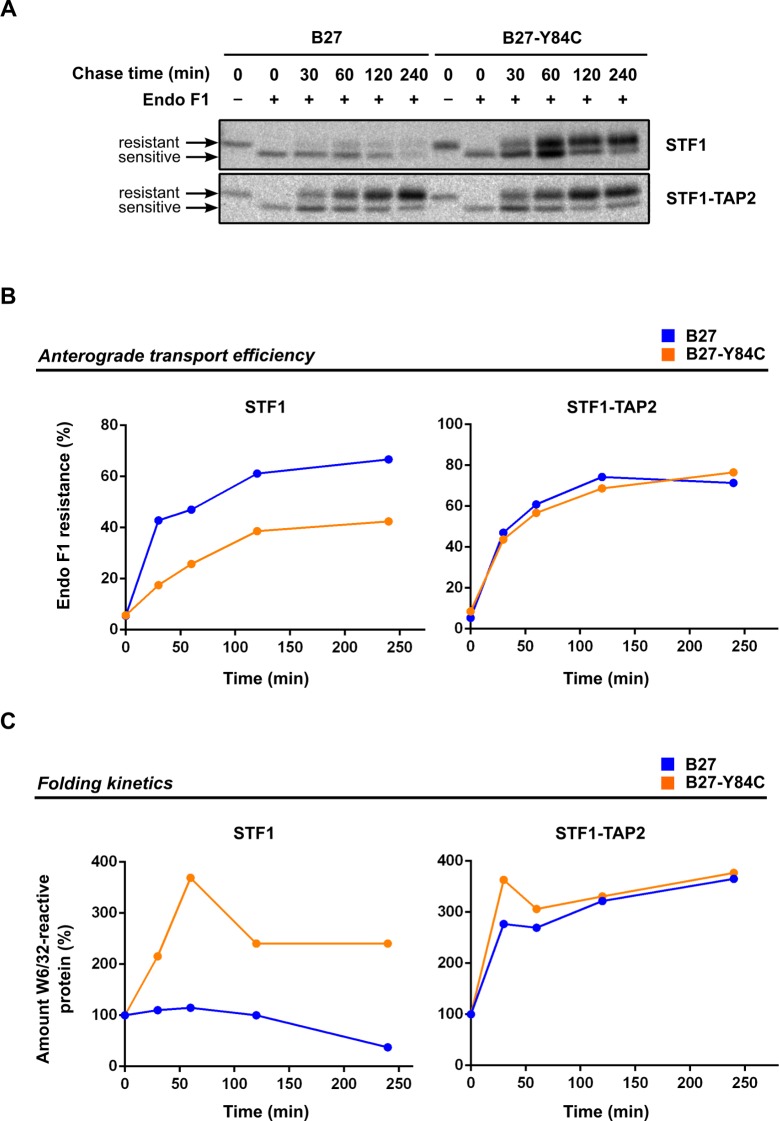
The disulfide mutant of HLA B*27:05 shows accelerated anterograde transport and a longer lifetime than the wild type form in TAP2-deficient cells. (A) Pulse-chase analysis and Endo F1 resistance. STF1 (upper panel) and STF1-TAP2 cells (lower panel) expressing HA-tagged wild type B*27:05 and B*27:05-Y84C were labeled with ^35^S-methionine/-cysteine and were chased for the times indicated. HA-tagged molecules were isolated from lysates by sequential immunoprecipitation using W6/32 and anti-HA antibodies, respectively. Where indicated, immunoisolates were treated with Endoglycosidase F1. (B) Quantification of Endo F1 resistance. The total band intensity was calculated for each lane, and the proportion of the Endo F1-resistant band was depicted as percentage of the total. The disulfide mutant B*27:05-Y84C reaches Endo F1 resistance faster than the wild type form in peptide-deficient STF1 cells (left), whereas in peptide-proficient STF1-TAP2 cells, both constructs show similar glycan maturation rates (right). (C) Folding kinetics calculated by W6/32 reactivity. The total amounts of protein (Endo F1-sensitive plus Endo F1-resistant bands) were calculated for each lane, and the intensities were depicted as percentage of the total intensity of the band from time point zero. In peptide-deficient STF1 cells, the amount of wild type B*27:05 remains stable during the first hour of chase and starts to decline rapidly, whereas the amount of B*27:05-Y84C increases steadily within the first hour, and degradation of this molecule occurs much more slowly (left). In peptide-proficient STF1-TAP2 cells, the production and folding of wild type B*27:05 and B*27:05-Y84C are comparable (right).

The surprising finding that the increased cell surface level of B*27:05-Y84C in STF1 cells was not influenced by endocytosis kinetics then prompted us to investigate the folding and anterograde intracellular transport kinetics of both proteins. For this, we followed the biochemical maturation of the HA-tagged wild type B*27:05 and B*27:05-Y84C along the secretory pathway in peptide-deficient STF1 and peptide-proficient STF1-TAP2 cells. Fig 4A shows the results of pulse-chase analysis and Endo F1 digest, revealing that a small proportion of the wild type B*27:05 molecules progressed beyond the *cis*-Golgi within 60 min after synthesis (determined by the Endo F1-resistant bands) in peptide-deficient STF1 cells (upper panel, left), but within the next two hours (time points 120 and 240 min) the protein nearly disappeared, indicating rapid degradation (Fig 4C, left). In contrast, almost all of newly synthesized B*27:05-Y84C has already travelled beyond the *cis*-Golgi within 30 min after chase in STF1 cells (Fig 4A, upper panel, right). The increasing amount of W6/32-reactive protein indicates that the folding of B*27:05-Y84C continued until the end of the 60 min chase time point, and both the Endo F1-resistant and the Endo F1-sensitive forms which could be detected throughout the chase period indicated a longer lifetime for the disulfide mutant than for the wild type form (Fig 4C, left). In peptide-proficient STF1-TAP2 cells, however, both constructs trafficked rapidly towards the cell surface and accumulated in their Endo F1-resistant forms during the chase period (Fig 4A, lower panel, and Fig 4B, right). The steady increase of a W6/32-reactive signal indicates that the folding of both proteins continued throughout the chase (Fig 4C, right). These results support the previous observations that B*27:05-Y84C can escape protein quality control in the early secretory pathway, whereas wild type B*27:05 is degraded intracellularly when optimal peptides are not present.

### In the absence of optimal peptide, the disulfide mutant of HLA-B*27:05 is more thermostable and structurally more ordered than the wild type

**Fig 5:**
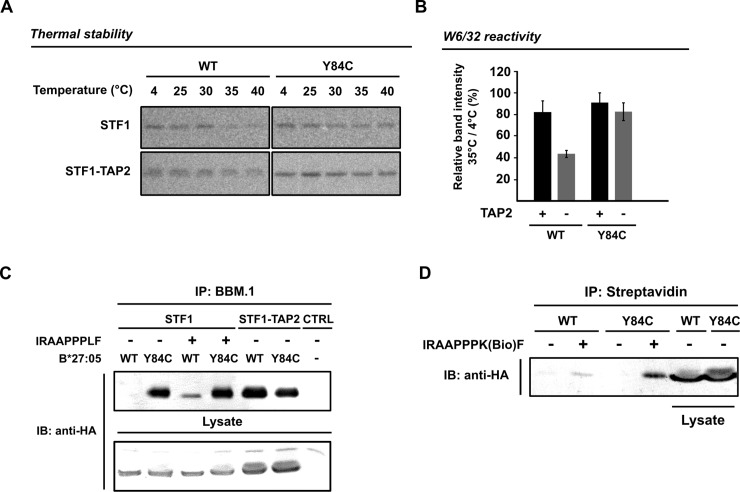
B*27:05-Y84C is more resistant to thermal denaturation and structurally more ordered in the peptide-receptive state. (A) Thermal stability assay. STF1 (upper panel) and STF1-TAP2 cells (lower panel) expressing HA-tagged wild type B*27:05 and B*27:05-Y84C were pulse-labeled with ^35^S-methionine/-cysteine for 7 min and then lysed. Detergent lysates were incubated at the temperatures indicated for 10 min prior to sequential immunoprecipitation with W6/32 and anti-HA, respectively. (B) Quantification of thermal stability. Band intensities from lanes representing 4 and 35°C were calculated for the individual samples. Thermal stability of the constructs is calculated and displayed in a bar chart as the ratio of the 35°C signal over the 4°C signal from W6/32 immunoprecipitation (SEM, n = 3). Wild type B*27:05 shows a decrease in thermal stability in the absence of TAP2 (left) whereas B*27:05-Y84C is equally stable in STF1 and STF1-TAP2 cells. (C) *β*_2_m affinity measurement. Human β_2_m was immunoprecipitated from cell lysates using BBM.1 antibody. Co-immunoprecipitated B*27:05 heavy chains were identified by western blotting using HA antibody (upper row). Where indicated, a high-affinity ligand for B*27:05 (IRAAPPPLF) was added to the lysate prior to immunoprecipitation. STF1-TAP2 cells were used as a positive control for BBM.1 immunoprecipitation. Last lane (CTRL) indicates STF1 cells that do not express B*27:05. Equal amount of lysate (2% of total) was loaded on a separate gel as a loading control (lower row). B*27:05 heavy chains were detected using anti-HA in immunoblotting (IB). (D) Peptide binding to B*27:05. Biotinylated IRAAPPPKF peptide was added to the cells (where indicated) at the point of lysis, and complexed proteins were isolated using streptavidin-conjugated agarose beads. Associated B*27:05 heavy chains were detected using anti-HA antibody in immunoblotting. Last two lanes are loading controls from the lysates (2% of total).

We next sought to understand the molecular mechanism underlying the superior ER-surface transport competence of HA-tagged B*27:05-Y84C in cells where TAP complex is not functional. We hypothesized that one reason why B*27:05-Y84C could escape quality control checkpoints in the early secretory pathway might lie in its ability to preserve a folded conformation in the absence of TAP-dependent peptides. To compare the relative conformational stability of wild type B*27:05 and B*27:05-Y84C, we determined the amounts of W6/32-reactive proteins in cell lysates that were gradually heated up to 40°C. It is known that the amount of W6/32-positive HLA-B decreases drastically in heated lysates when tapasin is absent and, hence, peptide loading onto HLA-B is impaired [34]. We thus hypothesized that B*27:05-Y84C would display a different heat sensitivity pattern that the wild type form, especially in TAP2-deficient STF1 cells. In peptide-proficient STF1-TAP2 cells, both constructs remained rather stable up to a temperature of 40°C confirming that the TAP-mediated supply of optimal peptides supported the folded conformation of HLA molecules in detergent lysates (Fig 5A, lower panel). In peptide-deficient STF1 cells, however, the amount of W6/32-reactive wild type B*27:05 remained stable only until 30°C and started to drastically decrease from 35°C onwards, whereas higher amounts of B*27:05-Y84C were detected in lysates heated up to 35 and 40°C (Fig 5A, upper panel). To be able to illustrate the difference in the thermal stabilities of both constructs in TAP2-deficient STF1 cells more clearly, we calculated the 35°C / 4°C signal ratio, which clearly demonstrated the higher thermal stability of the disulfide mutant *versus* wild type B*27:05 at 35°C in TAP2-deficient STF1 cells (Fig 5B, left). In peptide-proficient STF1-TAP2 cells, both constructs showed comparable thermal stabilities at 35°C (Fig 5B, right). To determine if the higher thermal stability of the heterodimer is due to the increased affinity between the B*27:05-Y84C heavy chain and *β*_2_m, we then isolated and quantified the amount of *β*_2_m -associated B*27:05 heavy chains from cell lysates. In TAP-deficient STF1 cells, immunoprecipitation with human *β*_2_m-specific monoclonal antibody BBM.1 yielded plenty of B*27:05-Y84C heavy chains while amount of BBM.1-reactive wild type B*27:05 complexes remained below detection level (Fig 5C). We were able to recover low amounts of wild type B*27:05 heavy chains only in the presence of B*27:05-specific peptide (IRAAPPPLF, IF9) during lysis. As expected, similar amounts of wild type and mutant B*27:05 heavy chains were isolated from TAP-proficient cells (Fig 5C, lanes STF1-TAP2), indicating a reduction in the *β*_2_m *–*affinity of the wild type B*27:05 in the absence of high-affinity peptide ligands. We did not observe an increase in the amount of BBM.1-reactive B*27:05-Y84C heterodimers after having added the high affinity IF9 peptide to the lysates (compare lanes 2 and 4 in Fig 5C). This prompted us to investigate if the B*27:05-Y84C was able to bind the peptide in the first place. We therefore isolated the peptide-bound B*27:05 molecules through immunoprecipitation of the B*27:05-biotinylated IF9 complexes with streptavidin. We recovered considerably more B*27:05-Y84C bound to biotinylated IF9 than the wild type (Fig 5D), indicating the ability of the Y84C mutant to preserve a stable and peptide-receptive conformation in TAP-deficient cells.

## Discussion

Human and mouse cells are capable of recognizing MHC class I molecules that have failed to load high-affinity peptides and of preventing them from reaching the cell surface in order to avoid binding of extracellular peptides. In search of a molecular explanation for the differences between class I molecules loaded with high-affinity peptides and suboptimally loaded class I molecules bound to low-affinity peptides, we have discovered a region in the class I heavy chain that is most likely targeted by the cellular quality control systems. This region, comprised of the amino acids that form the F pocket, becomes highly flexible when a peptide fails to interact with the heavy chain via its C terminus [9,10]. In the presence of a high-affinity peptide, the flexibility of the F pocket region is reduced by the increasing number of hydrophobic interactions as well as hydrogen bonds between the side chains of the peptide and the F pocket residues [35]. We demonstrated that the immobilization of the F pocket by fixing the ends of the α_1_ and α_2_−1 helices was sufficient to stabilize the murine MHC class I allotype H-2K^b^ (as well as another murine allotype H-2D^b^, unpublished data) such that it could escape ER quality control [13]. Since the residues involved in this artificial fixation (tyrosine 84 and alanine 139) are highly conserved for classical MHC Class I molecules among species, we decided to investigate whether the disulfide mutation would have the same effect on HLA-B*27:05, a human class I subtype that was previously shown to have low intrinsic stability [36] and tendency to form unusual heavy chain configurations [37].

First, we used comparative MD simulations of HLA-B*27:05, we showed reduced flexibility of the residues in the F pocket for the empty Y84C mutant similar to that of the peptide-bound HLA-B*27:05. Due to high computational demand, MD simulations are limited in predicting and analyzing conformational changes in proteins only within a nanosecond-timescale, which does not reflect all possible alterations that are taking place in class I molecules, which have lost their peptide ligands. Nevertheless, our results are consistent with previous MD simulations (nano/microsecond-timescale) of human and murine allotypes [38–40] and suggest that the 84-139 disulfide bond restrains the F-pocket flexibility similar to the binding of the C-terminal residue of the bound peptide.

Next, we showed that cell surface expression of HA-tagged wild type HLA-B*27:05 is dependent on peptides delivered to the ER via the TAP transporter since it is intracellulary retained in TAP2-deficient STF1 cells (**Fig 2**). The steady state levels were restored by the introduction of the *TAP2* gene (STF1-TAP2 cells), indicating that the reduced cell surface expression of the wild type protein is due to the lack of high-affinity peptides in the ER. The disulfide mutant B*27:05-Y84C, however, was readily detected at the surface of STF1 cells, indicating the ability of this molecule to bypass intracellular retention mechanisms that apply to empty or suboptimally loaded MHC class I molecules. This observation reinforces the suggestion that a general mechanism exists in cells that surveys the flexibility of the helices in the peptide binding domain of class I heavy chains as an indicator of optimal peptide selection. Similar levels of wild type B*27:05 and B*27:05-Y84C at the surface of STF1-TAP2 cells (**Fig 2**) and pull-down experiment using B*27:05-specific peptide (**Fig 5D**) imply that the disulfide mutant can bind peptides and can present them at the cell surface. Despite its ability to bind high-affinity peptide (**Figs 5D** and **S2D**), B*27:05-Y84C disappeared more quickly from the surface of TAP2-proficient cells than the wild type B*27:05 (**Fig 3**). Loss of the conformation recognized by W6/32 antibody in B*27:05-Y84C despite the presence of optimal peptides is supported by our observation that lesser amounts of *β*_2_m-bound Y84C heavy chains can be recovered from TAP-proficient cells (**Fig 5C,** compare lanes 4 and 6). This contrasts with our previous biochemical and cell biological analyses that show that the disulfide mutant of H-2K^b^ is equally or only slightly less stable than the wild type in peptide-bound form [13]. It is plausible to suggest that the hydrogen bonds between peptide C terminus and tyrosine at position 84 contributes differentially to the stability of the trimers among allotypes and peptides, but their absence reduces the overall stability of the peptide-MHC I complex in general. Other residues involved in interactions with the peptide (such as N97) might also drive B*27:05 to accomodate a more open conformation of the F pocket, [41,42] which is not possible for B*27:05-Y84C. Since the relative molecular stability of B*27:05-Y84C is much higher than the wild type in TAP-deficient cells (higher *β*_2_m affinity and ability to bind peptides, **Figs 5 and S2D**), it can be speculated that the mutant attains a different (and probably much more stable) conformation in the ER when it is devoid of peptide. This is supported by our finding that the B*27:05-Y84C mutant, when newly synthesized, is more resistant to thermal denaturation (loss of W6/32 epitope) than wild type B*27:05 (**Fig 5A**) and that it continues to gain increasing recognition by W6/32 during its folding in STF1 cells **(Fig 4)**.

Surprisingly, the improvements in the binding strength between the heavy chain and *β*_2_m via C84-C139 disulfide linkage do not contribute to the lifetime of B*27:05-Y84C at the cell surface. This interesting observation helps us understand the consequences of the changes in the conformational states of class I molecules during their transport inside the cell and their residence at the cell surface. Combined with our previous observations from disulfide mutations from murine class I allotypes H-2K^b^ and H-2D^b^, we propose that preservation of the heavy chain-*β*_2_m heterodimer is sufficient for class I molecules to exit early secretory pathway. This rule applies to HLA-B*27:05, even though this particular class I molecule was previously shown to have an exceptionally low affinity for *β*_2_m [36]. HLA-B*27:05 was also shown to require relatively long time for folding [43–45]. Our intracellular transport studies have revealed that the stably folded state of B*27:05-Y84C manifests itself in a faster maturation rate of the protein inside the cell (**Fig 4**). In the same cells, B*27:05-Y84C is increasingly recognized by W6/32 within the first hour of chase, suggesting also slow folding but more efficient ER exit. The slight decrease of W6/32-reactive amounts of B*27:05-Y84C after two hours of chase (**Fig 4C**, left) are possibly due to the endocytic degradation of the molecules that have reached the cell surface. The drastic reduction in the amount of W6/32-reactive wild type B*27:05 after two hours of chase in TAP2-deficient STF1 cells is on the other hand most likely due to ER-associated degradation (ERAD) in the absence of peptide [46]. The lack of HA- or HC-10-positive wild type B*27:05 in TAP-deficient cell line STF1 (**S2C Fig**) also confirms the requirement of cell surface transport of B*27:05 for the generation of *β*_2_m-free heavy chains and disulfide linked structures arising from them [36,37].

In cells that lack components of the peptide loading complex, class I molecules are retained in the early secretory pathway [43], and low-stability heterodimers and trimers that have escaped early quality control steps are removed from the cell surface. We observe the outcome of the cell surface quality control mechanism by the disappearance of peptide-receptive or suboptimally loaded heterodimers from the surface that were brought up by incubating cells at low temperature [33,47]. We suggest that this quality control mechanism at the cell surface surveys primarily the binding strength of class I heavy chain and *β*_2_m, rather than the occupancy of class I molecules with peptides. Indeed, surface lifetimes of both wild type and disulfide mutant of B*27:05 were restored in STF1 cells when the BFA decay was performed at 25°C (**S2B Fig**). To our surprise, the enhanced structural stability of the peptide-receptive heterodimers of B*27:05-Y84C (**Fig 5C**) is insufficient in rescuing the mutant from endocytic degradation. This suggests that the Y84C-heterodimers dissociate shortly after their arrival at the cell surface. Supporting this assumption, we observe elevated levels of HC-10 positive heavy chains of B*27:05-Y84C at the surface of both STF1 cells (**S2C Fig**). The behaviour of B*27:05-Y84C in TAP-deficient cells might be considered comparable to the phenotype of AS-related B*27:05 subtypes in conjunction with non-synonymous *ERAP1* polymorphisms [48–51]. While B*27:05 fails to exit the early secretory pathway in the absence of peptides dependent on TAP, it should able to breach the first step of quality control and arrive at the surface when ERAP1 function is impaired. In line with this hypothesis, N-terminally elongated 11–13 mer peptides were eluted from B*27:05 in an *ERAP1*-silenced model system [52], underlining the ability of this class I subtype to bind non-canonical peptide motifs [53]. Several independent studies showed that the formation of HLA-B27 free heavy chains at the surface is highly susceptible to changes in ERAP1 activity (achieved by silencing of ERAAP or by the overexpression of the wild type or mutants or chemical inhibition of the enzyme) in model cell lines as well as in cells from AS-patients carrying ERAP1 polymorphisms [54–56]. Not all ERAP polymorphisms that are found in AS-patients result in increased levels of *β*_2_m-free B27 heavy chains [56], although functional impairment of this enzyme clearly alters the pool of peptides presented on HLA-B27 [48–52]. This implicates the requirement of an additional factor that controls the release of suboptimally loaded B27 molecules to the cell surface. In some instances, loss of ERAP1 activity leads to an increase in *β*_2_m-free B*27:05 in *ERAP1* knock-down cell models, while surface HC-10 levels of other tested HLA-B allotypes remained unchanged [54,55]. Those HC-10 molecules are most likely to be removed from the cell surface efficiently, however, they can possibly be stabilized against endocytic degradation by the formation of an intermolecular disulfide bond, mediated by the unpaired cysteine at position 67. When formed at the surface, such heavy chain dimers of B*27:05 can react with killer cell immunoglobulin-like receptors (KIR) in a peptide-independent fashion [57] and promote differentiation of pathogenic Th17 cells expressing the particular B*27:05-homodimer reactive KIR-3DL2 [58]. Empty B*27:05-Y84C at the surface of antigen presenting cells or multimers made of recombinant B*27:05-Y84C can be a useful tool for the detection and functional analyses of such alloreactive cells in patients.

Our study confirms the presence of a quality control mechanism in the early secretory pathway that surveys the flexibility of the F pocket of class I molecules in general. The artificially stabilized mutants of human and murine MHC molecules are useful tools to delineate protein folding and monitoring in cells. It is also noteworthy to mention that the disulfide mutants of class I are potentially powerful tools for diagnostic and therapeutic approaches involving MHC-multimers and *in vitro* T-cell activation.

## Supporting information

S1 Fig HLA cell surface expression after TAP2 reconstitution in STF1 cells. (A) TAP2 expression is restored in STF-TAP2 cells. The TAP2 gene was introduced into STF1 cells by viral transduction (generating STF1-TAP2 cells), and the expression of TAP2 was verified western blotting. Lysates of STF1 and STF-TAP2 cells were probed for TAP2 with the monoclonal antibody 429.3. Full length TAP2 could only be detected in STF-TAP2 cells migrating slightly more slowly than the 72 kDa band of the molecular marker. Calnexin was used as a loading control (upper band at 90 kDa). The asterisk marks an unspecific band detected in both lysates.
(B) TAP2 is fully functional in STF1-TAP2 cells. TAP function was confirmed by measuring the surface levels of endogenous HLA levels by using the monoclonal anti-HLA antibody, W6/32 and anti-mouse IgG conjugated to AlexaFluo-488 in flow cytometry. In peptide-deficient STF1 cells, only low cell surface expression of endogenous HLA molecules could be detected (dashed line), in peptide-proficient STF1-TAP2 cells, however, the surface expression was strongly enhanced (solid line) confirming functionality of the reconstituted TAP transporter.
(C) Cell surface expression of B*27:05 and B*27:05-Y84C. Cells were stained with W6/32 and anti-mouse IgG conjugated with AlexaFluor-488 and subjected to flow cytometry. Surface signal intensities from B*27:05 (blue) and B*27:05-Y84C (orange) are displayed as histograms. Grey lines indicate cells that were stained only with the secondary antibody.
(D) The scatter plot (mean ± standard deviation, n = 3) shows individual cell surface W6/32 measurements in STF1 and STF-TAP2 cells (black dots). In TAP2-deficient STF1 cells, surface expression of B*27:05-Y84C was about three times higher than for the wild type construct (left) whereas in TAP2-proficient cells, both constructs showed comparable cell surface expression (right).
(TIF)

S2 Fig Surface lifetimes of wild type and disulfide mutant of HLA B*27:05 can be rescued at the cell surface of TAP2-deficient cells at 25°C. (A) Wild type B*27:05 reaches the cell surface of TAP2-deficient cells at 25°C. Peptide-deficient STF1 cells expressing wild type B*27:05 were kept at 25 and 37°C, respectively, stained with anti-HA and anti-mouse IgG conjugated with AlexaFluor-488, and subjected to flow cytometry. Wild type B*27:05 shows a much higher cell surface expression at 25 (blue line) than at 37°C (orange line). The grey curve in both histograms shows the background signal without primary antibody. Quantification of surface signals obtained at 25°C (blue) and 37°C (black, set to one) revealed a 4-fold increase in surface levels of wild type B*27:05 (scatter plot with mean ± standard deviation, right).
(B) Averaged BFA decay from the cell surface at 25°C. STF1 cells were kept at 25°C and surface levels of B*27:05 and B*27:05-Y84C were detected by staining STF1 cells with anti-HA. Cells were harvested and stained at the times indicated representing the duration of treatment with Brefeldin A. The graph shows the cell surface levels normalized to the values detected at time point zero (SEM, n = 4), which was set to 100% with the following values depicted as its percentage. Both constructs show similar residence times at the cell surface when incubated at 25°C.
(C) B*27:05 free heavy chains on the surface of TAP-deficient cells. Scatter plot (mean ± standard deviation, n = 2,4,4) shows the levels of class I free heavy chains detected by HC-10 antibody at the surface of STF1, STF1-B*27:05 and STF1-B*27:05-Y84C cells at 37°C, respectively. Acquired staining intensities from individual experiments were normalized to wild type B*27:05 levels. B*27:05-Y84C reveals approximately 4-fold higher more free heavy chains that the wild type protein.
(D) Peptide binding to B*27:05 at the cell surface. STF1 cells expressing either B*27:05-WT or B*27:05-Y84C were incubated with 20 **μ**M of the B*27:05-specific peptide IRAAPPPLF overnight (black bars). Amount of B*27:05 molecules were detected with anti-HA antibody and displayed in comparison with the samples without peptide addition (grey bars). IRAAPPPLF can bind and stabilize B*27:05-Y84C molecules that have reached cell surface whereas surface levels of B*27:05-WT cannot be improved by the peptide.
(TIF)
