# Correction: Distinct mechanisms survey the structural integrity of HLA-B*27:05 intracellularly and at the surface

**DOI:** 10.1371/journal.pone.0203092

**Published:** 2018-08-27

**Authors:** Zeynep Hein, Britta Borchert, Esam Tolba Abualrous, Sebastian Springer

There is an error in the XML that is causing the third author’s name to be indexed incorrectly. The name should be indexed as: Abualrous ET. The correct citation is: Hein Z, Borchert B, Abualrous ET, Springer S (2018) Distinct mechanisms survey the structural integrity of HLA-B*27:05 intracellularly and at the surface. PLoS ONE 13(8): e0200811. https://doi.org/10.1371/journal.pone.0200811
