# Supplementary figures and images for: Distinct mechanisms survey the structural integrity of HLA-B*27:05 intracellularly and at the surface
Source: PLoS One. 2018 Aug 2;13(8):e0200811. doi: 10.1371/journal.pone.0200811 (PMC6071996; doi:10.1371/journal.pone.0200811)

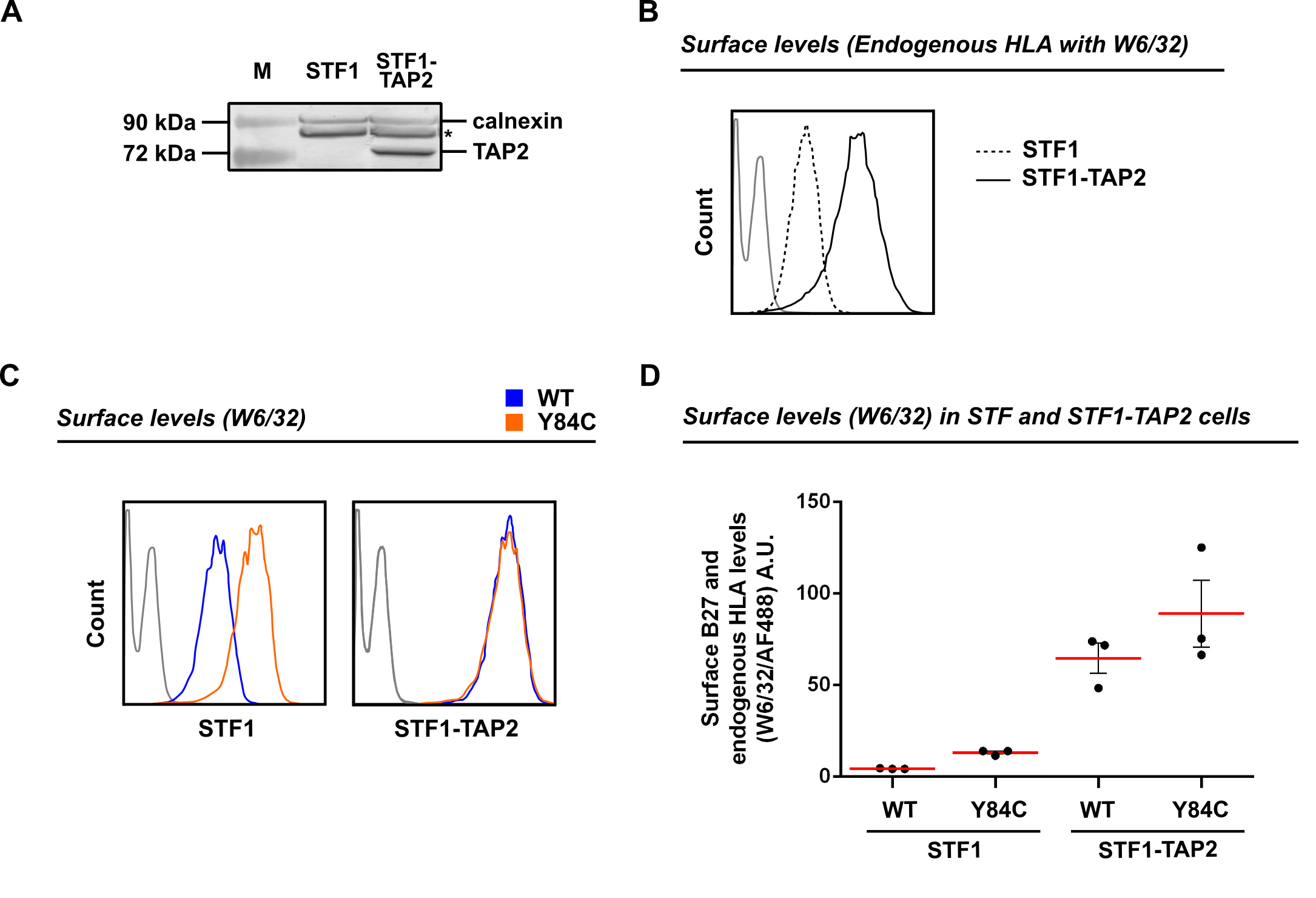

Supplement: S1 Fig — (A) TAP2 expression is restored in STF-TAP2 cells. The TAP2 gene was introduced into STF1 cells by viral transduction (generating STF1-TAP2 cells), and the expression of TAP2 was verified western blotting. Lysates of STF1 and STF-TAP2 cells were probed for TAP2 with the monoclonal antibody 429.3. Full length TAP2 could only be detected in STF-TAP2 cells migrating slightly more slowly than the 72 kDa band of the molecular marker. Calnexin was used as a loading control (upper band at 90 kDa). The asterisk marks an unspecific band detected in both lysates. (B) TAP2 is fully functional in STF1-TAP2 cells. TAP function was confirmed by measuring the surface levels of endogenous HLA levels by using the monoclonal anti-HLA antibody, W6/32 and anti-mouse IgG conjugated to AlexaFluo-488 in flow cytometry. In peptide-deficient STF1 cells, only low cell surface expression of endogenous HLA molecules could be detected (dashed line), in peptide-proficient STF1-TAP2 cells, however, the surface expression was strongly enhanced (solid line) confirming functionality of the reconstituted TAP transporter. (C) Cell surface expression of B*27:05 and B*27:05-Y84C. Cells were stained with W6/32 and anti-mouse IgG conjugated with AlexaFluor-488 and subjected to flow cytometry. Surface signal intensities from B*27:05 (blue) and B*27:05-Y84C (orange) are displayed as histograms. Grey lines indicate cells that were stained only with the secondary antibody. (D) The scatter plot (mean ± standard deviation, n = 3) shows individual cell surface W6/32 measurements in STF1 and STF-TAP2 cells (black dots). In TAP2-deficient STF1 cells, surface expression of B*27:05-Y84C was about three times higher than for the wild type construct (left) whereas in TAP2-proficient cells, both constructs showed comparable cell surface expression (right). (TIF) [file pone.0200811.s001.tif]

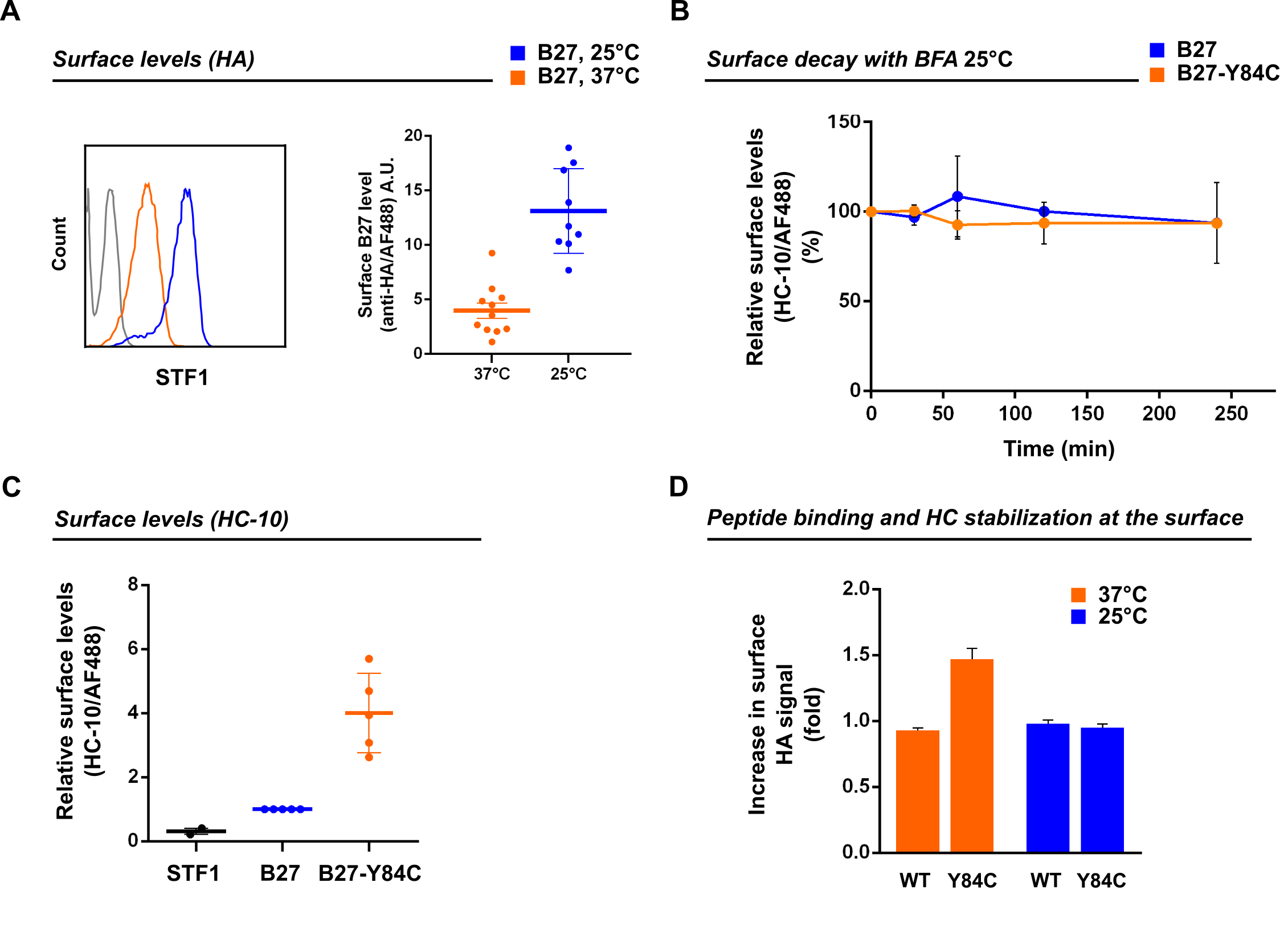

Supplement: S2 Fig — (A) Wild type B*27:05 reaches the cell surface of TAP2-deficient cells at 25°C. Peptide-deficient STF1 cells expressing wild type B*27:05 were kept at 25 and 37°C, respectively, stained with anti-HA and anti-mouse IgG conjugated with AlexaFluor-488, and subjected to flow cytometry. Wild type B*27:05 shows a much higher cell surface expression at 25 (blue line) than at 37°C (orange line). The grey curve in both histograms shows the background signal without primary antibody. Quantification of surface signals obtained at 25°C (blue) and 37°C (black, set to one) revealed a 4-fold increase in surface levels of wild type B*27:05 (scatter plot with mean ± standard deviation, right). (B) Averaged BFA decay from the cell surface at 25°C. STF1 cells were kept at 25°C and surface levels of B*27:05 and B*27:05-Y84C were detected by staining STF1 cells with anti-HA. Cells were harvested and stained at the times indicated representing the duration of treatment with Brefeldin A. The graph shows the cell surface levels normalized to the values detected at time point zero (SEM, n = 4), which was set to 100% with the following values depicted as its percentage. Both constructs show similar residence times at the cell surface when incubated at 25°C. (C) B*27:05 free heavy chains on the surface of TAP-deficient cells. Scatter plot (mean ± standard deviation, n = 2,4,4) shows the levels of class I free heavy chains detected by HC-10 antibody at the surface of STF1, STF1-B*27:05 and STF1-B*27:05-Y84C cells at 37°C, respectively. Acquired staining intensities from individual experiments were normalized to wild type B*27:05 levels. B*27:05-Y84C reveals approximately 4-fold higher more free heavy chains that the wild type protein. (D) Peptide binding to B*27:05 at the cell surface. STF1 cells expressing either B*27:05-WT or B*27:05-Y84C were incubated with 20 μM of the B*27:05-specific peptide IRAAPPPLF overnight (black bars). Amount of B*27:05 molecules were detected with anti-HA antibody [file pone.0200811.s002.tif]
